# Heterogeneity of Loss Aversion in Pathological Gambling

**DOI:** 10.1007/s10899-015-9587-1

**Published:** 2015-12-28

**Authors:** Hideaki Takeuchi, Ryosaku Kawada, Kosuke Tsurumi, Naoto Yokoyama, Ariyoshi Takemura, Takuro Murao, Toshiya Murai, Hidehiko Takahashi

**Affiliations:** Department of Psychiatry, Kyoto University Graduate School of Medicine, 54 Shogoin-Kawahara-cho, Sakyo-ku, Kyoto 6068507 Japan

**Keywords:** Behavioral economics, Heterogeneity, Loss aversion, Pathological gambling, Personality traits

## Abstract

Pathological gambling (PG) is characterized by continual repeated gambling behavior despite negative consequences. PG is considered to be a disorder of altered decision-making under risk, and behavioral economics tools were utilized by studies on decision-making under risk. At the same time, PG was suggested to be a heterogeneous disorder in terms of personality traits as well as risk attitude. We aimed to examine the heterogeneity of PG in terms of loss aversion, which means that a loss is subjectively felt to be larger than the same amount of gain. Thirty-one male PG subjects and 26 male healthy control (HC) subjects underwent a behavioral economics task for estimation of loss aversion and personality traits assessment. Although loss aversion in PG subjects was not significantly different from that in HC subjects, distributions of loss aversion differed between PG and HC subjects. HC subjects were uniformly classified into three levels (low, middle, high) of loss aversion, whereas PG subjects were mostly classified into the two extremes, and few PG subjects were classified into the middle range. PG subjects with low and high loss aversion showed a significant difference in anxiety, excitement-seeking and craving intensity. Our study suggested that PG was a heterogeneous disorder in terms of loss aversion. This result might be useful for understanding cognitive and neurobiological mechanisms and the establishment of treatment strategies for PG.

## Introduction

Pathological gambling (PG) is a chronic psychiatric disorder characterized by continual repeated gambling behavior despite negative consequences. In addition to PG patients themselves, their families and workplaces sometimes face serious consequences. Although PG was classified by the Diagnostic and Statistical Manual of Mental Disorders, 4th Edition Text-Revision (DSM-IV-TR) (American Psychiatric Association 2000) as “Impulse-Control Disorder Not Elsewhere Classified”, according to the Diagnostic and Statistical Manual of Mental Disorders 5th Edition (DSM-5) (American Psychiatric Association 2013), it is classified into “Substance-Related and Addictive Disorders”.

Although PG is classified into the same category as substance-use addictions in DSM-5, it has a specific behavior and a different diagnostic criterion from substance-use addictions called “chasing”. Chasing is considered a continual gambling behavior in order to regain lost money and almost always results in a more miserable situation. Continual gambling despite continual defeat may be attributed to altered decision-making under risk. Recently, in order to clarify altered decision-making in neuropsychiatric disorders, tools of the behavioral economics field have been utilized in clinical psychiatry. One of the most influential and successful theories of decision-making under risk is the prospect theory (Kahneman and Tversky 1979). A core part of the prospect theory is the probability weight function, which is expressed by an inverse S-shaped curve. This function shows that higher probability tends to be underestimated and lower probability tends to be overestimated. Previous studies investigated a neural substrate (Hsu et al. 2009) and a molecular mechanism (Takahashi et al. 2010) of this function. Another core part of the prospect theory is the value function, which is expressed as a loose, concave slope in a gain area, and a steep, convex slope in a loss area. Generally, the susceptibility to loss is stronger than that to gain as, for example, few people try a gamble that has a 50–50 chance of winning and losing the same amount of money. This psychological trait is called loss aversion, meaning that a loss is felt to be larger subjectively than the same amount of gain, even if they are objectively equivalent. Loss aversion is expressed as a steeper slope in a loss area than that in a gain area. Previous studies investigating the neural substrate of loss aversion suggested that striatum (Chib et al. 2012; Tom et al. 2007) and amygdala (De Martino et al. 2010) are involved in this phenomenon, and loss aversion is modulated by thalamic norepinephrine transmissions (Takahashi et al. 2013).

Because PG patients tend to have risk-taking personality and show reckless decision-making (Slutske et al. 2005), the intuitive assumption is that PG patients would show diminished loss aversion, as seen in amygdala lesion patients (De Martino et al. 2010). On the other hand, a recent field study reported that internet problem gamblers tended to show loss aversive or risk aversive decision-making during chasing, pointing to a relationship between loss aversion and chasing (Xuan and Shaffer 2009). After losing money to gambling, PG patients appear to be chasing their losses (continue gambling) to get even. The value function, which is concave in a gain area and convex in a loss area, explains the typically observed pattern of risk-seeking for losses and risk aversion for gains. Furthermore, loss aversion as expressed by a steeper slope in a loss area exaggerates risk-seeking for losses and risk aversion for gains. A related cognitive bias concerned with loss aversion in money investment is the disposition effect, which refers to a bias of investors who tend to sell assets (risk aversion) when their price is rising, and keep them (risk-seeking) when their price is falling. Although this could be mainly explained by the fact that the value function is concave in a gain area and convex in a loss area, the loss aversion (a steeper slope of the value function in a loss area) could exaggerate this bias (Kahneman and Tversky 1979; Shefrin and Statman 1985). It is thought that the difficulty of selling assets when their price is falling (loss cut) is similar to the difficulty of deciding when to stop in losing gambles (chasing). Another study reported that PG patients in a late treatment stage showed greater loss aversion than healthy control (HC) subjects, whereas PG patients in an early treatment stage showed similar loss aversion to HC subjects (Giorgetta et al. 2014). Indeed, these previous studies suggested that the role of loss aversion in decision-making of PG is not so straightforward.

Previous studies indicated the existence of subtypes in PG on the basis of personality traits (Graham and Lowenfeld 1986; McCormick 1988; Moran 1970; Steel and Blaszczynski 1996; Zimmerman et al. 1985). The pathways model was advocated based on those previous studies (Blaszczynski and Nower 2002) and indicated two subtypes deeply related with personality traits. One group is called ‘emotionally vulnerable problem gamblers’. They are characterized by premorbid anxiety and/or depression, and have poor skills of coping and solving problems. The other group is called ‘antisocial impulsivist problem gamblers’. Their characteristic personality traits are high impulsivity and/or high excitement-seeking.

The effectiveness of drugs directed at these personality traits was examined for PG. The effects of antidepressant drugs, which are effective against anxiety and depression, have so far provided mixed results (Black et al. 2007; Blanco et al. 2002; Grant et al. 2003; Hollander et al. 2000; Kim et al. 2002; Saiz-Ruiz et al. 2005), and the effect of drugs utilized for attention deficit hyperactivity disorder (ADHD), a disorder related to impulsivity and excitement-seeking, for example, amphetamine (Zack and Poulos 2004) and modafinil (Smart et al. 2013), was also examined for PG, but a pharmacological treatment for PG remains to be established. This also suggests that PG is a heterogeneous disorder with personality traits.

Here, we aimed to examine loss aversion of PG using behavioral economics tools that can directly approach gambling behaviors and personality traits. We hypothesized that PG was heterogeneous in terms of loss aversion and that loss aversion would be associated with clinical scales and personality traits such as anxiety, depression, impulsivity and excitement-seeking.

## Materials and Methods

### Participants

The PG group consisted of 31 male patients who were referred to a treatment facility. The age-matched HC group consisted of 26 healthy male controls. All HC subjects were recruited from a local community. All PG subjects met the criteria for PG according to DSM-IV-TR, and PG symptoms were investigated using the Structured Clinical Interview for Pathological Gambling (SCI-PG) (Grant et al. 2004). Comorbid disorders were screened with the Structure Clinical Interview for DSM-IV-TR. The treatment facility is a residential facility where PG patients receive 12-step-based psychological therapy. All PG subjects were medication-free and participated after they had completed at least one cycle of 12-step-based intervention (about 1 month). HC subjects were also evaluated with the Structured Clinical Interview for DSM-IV-TR, and were found to have no history of any psychiatric disorders. All PG subjects were physically healthy at the time of the assessment. None of the subjects had a history of neurological injury or disease, severe medical disease, or illegal substance abuse that might affect brain function. Demographic data of all subjects were collected with respects to age, education level, and smoking status. Smoking status was evaluated with the Fagerström Test for Nicotine Dependence (FTND) (Heatherton et al. 1991). We assert that all procedures contributing to this work complied with the ethical standards of the relevant national and institutional committees on human experimentation and with the Helsinki Declaration of 1975, as revised in 2008, and ensure that subjects’ confidentiality was in no way breached. This study was approved by the Committee on Medical Ethics of University and was carried out in accordance with the Code of Ethics of the World Medical Association. After offering a complete description of the study, written informed consent was obtained from all subjects.

### Experimental Procedures

#### Clinical Assessment

We assessed gambling severity using the South Oaks Gambling Screen (SOGS) (Lesieur and Blume 1987). SOGS is a 16-item self-administered questionnaire, ranging from 0 to 20. A score of 5 or higher indicates a risk of pathological gambling. We assessed symptoms of craving, which is common to addictive disorders, using the Gambling Craving Scale (GACS) (Young and Wohl 2009). GACS is a 9-item self-administered questionnaire assessed with a 7-point scale. A higher score indicates more intense craving. Duration of illness and abstinence were determined by questioning the PG subjects. We assessed IQ using predicted IQ based on the Japanese Adult Reading Test (JART) short form (Matsuoka and Kim 2007).

#### Personality Assessment

We assessed personality traits using Revised NEO Personality Inventory (NEO-PI-R) (Costa and McCrae 1992). NEO-PI-R is based on the five-factor model of personality using 240 items. The five factors consist of neuroticism, extraversion, openness, agreeableness, and conscientiousness. Then, we used the score of the five factors and four subscales: anxiety, depression, impulsiveness, and excitement-seeking based on the pathways model (Blaszczynski and Nower 2002).

#### Risky Choice Task

The behavioral loss aversion parameter for each subject was determined based on a staircase procedure suggested by Tversky and Kahneman (Tversky and Kahneman 1992). We used a gambling task to measure the behavioral loss aversion parameter. This task was the same as that used in a previous study (Takahashi et al. 2013). The subjects were presented with options between a mixed gamble (gain-loss) and a “stay” option on a computer monitor. Each mixed gamble had a 50 % chance of losing a fixed amount of X and a 50 % chance of gaining Y. A “stay” option was described as a mixed gamble that had a 50 % chance of losing 0 yen and a 50 % chance of gaining 0 yen (i.e., getting 0 yen for sure). We used 4 different possible losses (−X): −2500 yen, −5000 yen, −10,000 yen, and −15,000 yen. In each trial, the subjects chose between the mixed gamble and the “stay” option. The relative position (left or right) of the two options was randomized to counterbalance for order effects. The subjects were instructed as follows: “Two options of a mixed gamble will be presented to you. Make a choice between the two options according to your preference by pressing the right or left button. There is no correct answer and no time limit. Once you make a choice, the next pair of options will be presented. Actual remuneration does not reflect the result of the task.”

Each time a choice was made between a mixed gamble and a “stay” option in a trial, the amount of possible gain Y in the next trial was adjusted and ten trials of mixed gambles with possible loss (−X) were iterated to successively narrow the range including the amount of possible gain to make up for a 50 % chance of losing X. That is, we used a titration method to ensure consistent choices of the subjects. The adjustments in the amount of Y were made in the following manner. The initial range of Y was set between 0.5 × X and 10 × X. The range was divided into thirds. The one-third and two-thirds intersecting points of the initial range were used as possible gain Y in trials 1 and 2. If the subjects accepted the mixed gamble of the two-thirds and rejected the one-third in trials 1 and 2, the middle third portion of the initial range was used as a range for trials 3 and 4. If the subjects accepted both mixed gambles of the thirds, the lower third part was then used as range. If the subjects rejected both mixed gambles of the thirds, the upper third part was then used. The new range was again divided into thirds and the same procedure was iterated until the subject completed trial 10. The mean of the final range was used for the amount of gain Y_final_ to make up for a 50 % chance of losing X. Once Y_final_ was estimated for a given loss (−X), the gambles with the next loss (−X) were chosen for the estimation, and so on. The order of X was randomized across the subjects.

The subjects performed the task in a silent room in the presence of an experimenter. The experimenter checked the attitude of the subjects toward the task in order to exclude those who did not engage in the task seriously, and confirmed that all the subjects completed the task in a desirable manner.

### Parameter Estimation and Statistical Analysis

The amount of gain Y_final_ to make up for the 50 % chance of losing X is expressed as Y_final_ = λ × X, where λ is a loss aversion parameter. This λ parameter is similar to the parameter in prospect theory but makes the common simplifying assumptions of a linear rather than a curvilinear value function, and identical decision weights for a 0.5 probability of a gain or loss. (Note that the decision weight need not be equal to 0.5, as long as it is assumed to be the same for the gain probability and the loss probability.) Based on previous literature, we confined the range of λ from 0.5 to 10 during the estimation procedure. A smaller value of λ (closer to 0.5) means less loss aversion (actually, loss-seeking) and a higher value (closer to 10) means more loss aversion. The loss aversion parameter λ was estimated by least-squares method. Statistical analyses of demographic data, clinical data, and the loss aversion parameter λ was performed using SPSS (SPSS 22 for Windows, SPSS Inc., Chicago, IL, USA).

## Results

### Clinical and Personality Traits Assessment Between PG and HC

Clinical and personality traits assessment are summarized in Table 1 and tested by *t* tests. PG subjects showed significantly lower educational level and predicted IQ based on JART, and higher scores of FTND and SOGS than HC subjects. As for the five factors of NEO-PI-R, PG subjects showed significantly higher scores of neuroticism and lower scores of conscientiousness (agreeableness was marginally significant) than HC subjects. The two groups did not differ in terms of extraversion and openness. As predicted, PG subjects showed significantly higher subscales of anxiety, depression, impulsiveness, and excitement-seeking than HC subjects.

**Table 1:** Clinical and psychometric characteristics of PG and HC subjects

Variable	PG subjects (n = 31) Mean ± SD	HC subjects (n = 26) Mean ± SD	*p* value
Age (years)	33.4 ± 7.5	34.8 ± 6.3	0.45
Education level (years)	13.2 ± 1.9	16.6 ± 1.4	<0.001
FTND	3.5 ± 2.1	0.6 ± 1.4	<0.001
Predicted IQ based on JART	99.1 ± 9.5	111.3 ± 6.1	<0.001
SOGS	13.5 ± 2.4	0.3 ± 1.0	<0.001
GACS	21.0 ± 7.2		
Duration of illness (years)	12.0 ± 7.2		
Abstinence (months)	6.7 ± 6.7		
NEO-PI-R			
Neuroticism	117.6 ± 23.7	90.9 ± 17.8	<0.001
Extraversion	102.8 ± 18.0	100.0 ± 20.1	0.56
Openness	107.2 ± 15.2	110.2 ± 11.4	0.42
Agreeableness	103.9 ± 17.5	111.2 ± 10.6	0.06
Conscientiousness	87.3 ± 24.8	109.3 ± 18.8	<0.001
Anxiety	21.2 ± 5.8	17.4 ± 4.2	<0.01
Depression	20.8 ± 5.6	14.3 ± 3.7	<0.001
Impulsiveness	19.7 ± 5.0	15.2 ± 3.4	<0.001
Excitement-seeking	18.0 ± 4.2	15.5 ± 4.1	<0.05

*FTND* Fagerström Test for Nicotine Dependence, *JART* Japanese Adult Reading Test, *SOGS* South Oaks Gambling Screen, *GACS* Gambling Craving Scale, *NEO-PI-R* Revised NEO Personality Inventory

### Loss Aversion Parameter Assessment Between PG and HC

**Fig. 1:**
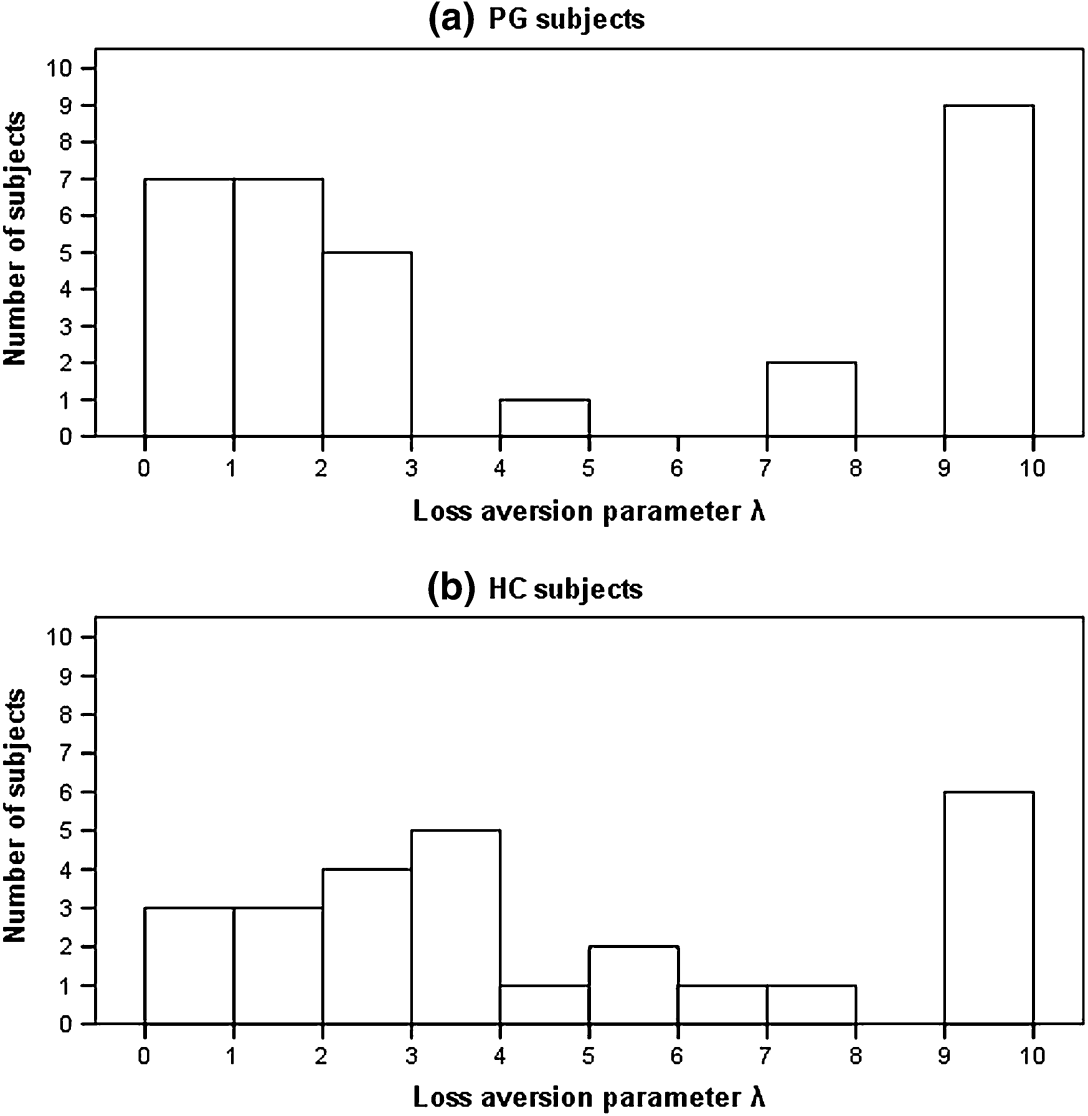
**a** Distribution of loss aversion parameter λ in PG subjects. **b** Distribution of loss aversion parameter λ in HC subjects

Loss aversion parameter λ in PG and HC subjects showed no significant correlation with age, education level, smoking status, and predicted IQ based on JART. The range of loss aversion parameter λ was 0.52–9.98 (median = 2.13) in PG subjects and 0.98–9.98 (median = 3.74) in HC subjects. Mann–Whitney’s U tests showed that PG and HC subjects did not differ in terms of loss aversion parameter λ (PG: mean = 4.40, SD = 3.95; HC: mean = 4.73, SD = 3.35; *p* = 0.37). Distributions of loss aversion parameter λ in PG and HC subjects are shown in Fig. 1. HC subjects showed a relatively uniform distribution, whereas PG subjects visually showed a biased distribution toward the two extremes, low and high. In order to investigate the distribution difference in greater detail, we divided the PG and HC subjects into three groups in terms of numerical value of loss aversion parameter λ, 0–3.33 (subjects with low loss aversion), 3.34–6.66 (subjects with middle loss aversion), and 6.67–10.00 (subjects with high loss aversion). Based on this, the HC group consisted of 12 subjects with low loss aversion, 7 subjects with middle loss aversion, and 7 subjects with high loss aversion. On the other hand, the PG group consisted of 19 subjects with low loss aversion, 1 subject with middle loss aversion, and 11 subjects with high loss aversion. There was a significant difference of distribution of loss aversion parameter λ between PG and HC subjects (Fisher’s exact test, *p* = 0.04).

### Characteristics of a Lower Loss Aversion Group and a Higher Loss Aversion Group in PG

According to the distribution of loss aversion in PG subjects, we compared the clinical scales and personality traits mentioned above (the five factors, and anxiety, depression, impulsiveness, excitement-seeking) between PG subjects with low loss aversion and PG subjects with high loss aversion. Clinical scales and NEO-PI-R scales of the two groups were tested by *t* tests and summarized in Table 2. They did not show any significant differences in terms of age, education level, FTND, predicted IQ based on JART, duration of illness, abstinence, and the five factors of NEO-PI-R. There were significant differences in terms of scores of GACS, anxiety and excitement-seeking between PG subjects with low loss aversion and PG subjects with high loss aversion. PG subjects with low loss aversion showed higher scores of GACS and excitement-seeking, and PG subjects with high loss aversion showed higher score of anxiety. Anxiety of PG subjects with low loss aversion was comparable to that of HC subjects (*p* = 0.22) and excitement-seeking of PG subjects with high loss aversion was similar to that of HC subjects (*p* = 0.45) as tested by *t* tests. We did not find any difference between HC subjects with low loss aversion and HC subjects with high loss aversion.

**Table 2:** Clinical and psychometric characteristics of PG subjects with low and high loss aversion

Variable	PG subjects with low loss aversion (n = 19) Mean ± SD	PG subjects with high loss aversion (n = 11) Mean ± SD	*p* value
SOGS	13.0 ± 2.6	14.5 ± 1.7	0.09
GACS	24.4 ± 7.3	16.1 ± 3.7	<0.01
NEO-PI-R			
Neuroticism	113.6 ± 26.4	122.0 ± 17.1	0.35
Extraversion	106.1 ± 18.1	97.5 ± 18.2	0.22
Openness	106.3 ± 14.7	107.8 ± 16.9	0.79
Agreeableness	104.8 ± 17.5	104.1 ± 18.2	0.91
Conscientiousness	89.3 ± 24.1	88.2 ± 23.4	0.91
Anxiety	19.3 ± 5.8	24.1 ± 4.5	<0.05
Depression	20.3 ± 5.8	20.6 ± 4.6	0.88
Impulsiveness	19.3 ± 5.6	19.9 ± 4.2	0.76
Excitement-seeking	20.0 ± 3.7	14.5 ± 2.7	<0.001

*SOGS* South Oaks Gambling Screen, *GACS* Gambling Craving Scale, *NEO-PI-R* Revised NEO Personality Inventory

## Discussion

PG subjects showed higher neuroticism, lower conscientiousness, higher anxiety, higher depression, higher impulsivity, and higher excitement-seeking than HC subjects. Interestingly, there was no significant difference in loss aversion parameters between PG and HC subjects. However, the distribution pattern of loss aversion parameters of PG subjects was differed significantly from that of HC subjects. Although HC subjects were uniformly classified into three levels (low, middle, high) of loss aversion, the majority of PG subjects were classified into either low or high level, and PG subjects virtually lacked the middle level. Therefore, we divided PG subjects into those with low loss aversion and those with high loss aversion. PG subjects with low loss aversion showed higher craving intensity and excitement-seeking than those with high loss aversion. PG subjects with high loss aversion showed higher anxiety than those with low loss aversion. Without efforts to clarify the difference in distributions between PG and HC subjects, it would seem at first glance that PG subjects uniformly have the same loss aversion and deviated personality traits based on simple patient-control comparison. Although excitement-seeking, anxiety, depression and impulsivity are generally higher in PG, PG subjects with low loss aversion showed high excitement-seeking and normal level anxiety, and those with high loss aversion showed high anxiety and normal level excitement-seeking.

We estimated loss aversion empirically using behavioral economics tools, which can assess risk attitude in real-life decision-making (Camerer 2004; Kahneman and Tversky 1984). This is the first study to demonstrate the heterogeneity of risk attitude in PG subjects and their relationships with personality traits. Since the pathways model was first proposed, knowledge of personality traits in relation to PG has been growing rapidly (Bonnaire et al. 2009; Ledgerwood and Petry 2006; Ledgerwood and Petry 2010; Stewart et al. 2008; Turner et al. 2008; Vachon and Bagby 2009). The current study could partly link the knowledge of loss aversion with personality traits in PG. PG with low loss aversion, high excitement-seeking and high craving intensity might partly coincide with ‘impulsivist gamblers’ as proposed by the pathways model, and those with high loss aversion and high anxiety might partly coincide with ‘emotionally vulnerable gamblers’. Previous studies have examined chasing from the view of risk attitude, with mixed results. One study reported that chasing reflected risk-taking attitude (Dickerson 1984), whereas a more recent study demonstrated that chasing reflected risk-aversive attitude (Xuan and Shaffer 2009). Identifying the heterogeneity in loss aversion from the perspective of risk attitude is very important in terms of understanding the cognitive and biological mechanisms of risk-taking behaviors.

The beneficial outcome of efforts to identify the heterogeneity in terms of loss aversion might also be useful for the development of treatment strategies for PG. PG subjects with low loss aversion demonstrate high excitement-seeking, and they may have features similar to the behavior of attention deficit hyperactivity disorder (ADHD) as proposed by the pathways model, and may possess the characteristic neural substrate. A previous study clarified that loss aversion has negative correlation with norepinephrine transporter (NET) density in the thalamus (Takahashi et al. 2013). Because the NET blocker atomoxetine is used in the treatment of ADHD, it might be worth considering the possible beneficial effect of NET blocker for reckless decision-making in PG with low loss aversion. On the other hand, pharmacological therapy that relieves anxiety, for example, anxiolytics and/or antidepressants, might be useful for PG with high loss aversion.

There are several limitations to our study. For ethical reasons, we decided not to use real money and gamble-related stimuli in abstinent and treatment-seeking PG patients. Concerns that such provocative stimuli might trigger craving or relapse were expressed by the treatment facility as well as the ethics committee. Although decision-making on hypothetical rewards does not necessarily reflect real life decision-making, validity of the results of experiments with hypothetical rewards has been reported (Locey et al. 2011). Because the current study was a cross-sectional study, it remains unclear whether the loss aversion parameter was modulated by treatment. A recent study showed that PG patients with a longer treatment history were more loss aversive than those with a shorter treatment history, implying that loss aversion could be modulated by treatment (Giorgetta et al. 2014). However, as that was also a cross-sectional study, it was difficult to actually determine the causal effect of treatment on loss aversion. In the future, in addition to cross-sectional research, longitudinal research to examine the treatment effect on loss aversion while paying attention to excitement-seeking, anxiety, and craving intensity at the individual level should be performed. We used only one behavioral task (loss aversion). Comprehensive measurements with various behavioral economics tools are recommended to enable us to fully understand the heterogeneity of altered decision-making in PG. Finally, all of the PG subjects in our study were undergoing treatment. It is estimated that just a small portion of PG patients is actually undergoing active treatment at any one time. Thus, any generalization of our findings needs to be approached with caution.

## Conclusion

We could examine the heterogeneity of PG in terms of loss aversion using behavioral economics tools. Although loss aversion in PG was not significantly different from that in HC, our findings suggest that PG is not a uniform disorder but rather a heterogeneous disorder with different risk attitudes. Future studies, especially neurobiological research, are needed to better understand the pathophysiology and to develop novel treatments for PG.
